# Proximate Composition, Phytochemicals, Phenolic Compounds, and Bioactive Characterization of *Mauritia flexuosa* L.f. Seeds

**DOI:** 10.3390/plants14152323

**Published:** 2025-07-27

**Authors:** Claudia Cristina Pérez Jaramillo, Liceth N. Cuéllar Álvarez, Walter Murillo Arango

**Affiliations:** 1 Grupo de Investigación en Productos Naturales, GIPRONUT, Universidad del Tolima, Ibagué 730006, Colombia; ccperezj@ut.edu.co; 2 Grupo de Investigación en Productos Naturales Amazónicos-GIPRONAZ, Facultad de Ciencias Básicas, Universidad de la Amazonia, Florencia 180001, Colombia

**Keywords:** canangucha, phenolic compounds, bioactive compounds, antihyperglycemic activity, antihypertensive activity, byproducts

## Abstract

*Mauritia flexuosa*, commonly known as “canangucha,” holds significant nutritional and economic value in the Amazon region. While its pulp is widely utilized in local food products, the seed or kernel is largely underutilized. This study investigated the proximal and phytochemical composition of *M. flexuosa*, alongside its biological properties, specifically focusing on the hypoglycemic activity of an ethanolic extract from *M. flexuosa* seeds (MFSs). Proximal analysis revealed that MFSs are a notable source of crude fiber (28.4%) and a moderate source of protein (9.1%). Phytochemical screening indicated a high total polyphenol content (123.4 mg gallic acid equivalents/100 mg dry weight) and substantial antiradical capacity against the ABTS radical (IC_50_ = 171.86 µg/mL). Notably, MFS ethanolic extracts exhibited significant in vitro antihyperglycemic activity via inhibiting α-amylase and α-glucosidase enzymes, demonstrating comparable inhibition to acarbose at higher concentrations. This hypoglycemic effect was further corroborated in an in vivo rat model with induced diabetes, where the administration of 100 mg/kg of MFS ethanolic extract significantly reduced blood glucose levels compared to the diabetic control group (*p* < 0.05). A moderate antihypertensive effect was observed at a concentration of 150 mg/kg, correlating with ACE inhibition. High-performance liquid chromatography–mass spectrometry (UHPLC-ESI-HRMS) analysis of the seed extract identified phenolic compounds including ellagic, p-coumaric, and chlorogenic acids, as well as flavonoids such as quercetin, myricetin, and epicatechin. This study provides the first evidence of the hypoglycemic activity of MFSs, offering valuable insights into their phytochemistry and potential therapeutic applications.

## 1. Introduction

*Mauritia flexuosa* (MF), commonly known as the buriti palm, moriche palm, or canangucha, is a species native to the Amazon basin that has garnered attention due to its diverse bioactive properties, supported by both ethnobotanical knowledge and emerging scientific studies [1]. Like many other species in the Amazon, MF is a source of phytocompounds, including phenolic compounds, flavonoids, tannins, carotenes, tocopherols, phytosterols, and other bioactive molecules that play a fundamental role in its pharmacological effects [2]. In the Amazonian forest, the canangucha fruit exhibits two ecotypes—I (elliptical) and II (round)—and studies analyzing its biomass distribution have found that its total biomass comprises 15.6% mesocarp pulp, 19.2% pericarp, and a substantial 65.2% seed or kernel [1].

MF pulp, the most utilized part of the fruit, is rich in oil, carotenoids, polyphenols, tocopherols, and ascorbic acid, which have been associated with antioxidant, antithrombotic, antiplatelet, and anti-inflammatory activities. Pentacyclic triterpenoids isolated from the fruit have shown significant bioactivity, inhibiting key inflammatory processes and activating antioxidant enzymes [3,4]. Moreover, MF exhibits various pharmacological effects, including hypolipidemic, photoprotective, antiaggregant, antimicrobial, and antitumor activities, highlighting its potential as a functional food and in pharmaceutical applications [4]. Although there is no direct evidence of the antidiabetic and antihypertensive potential of *M. flexuosa* seeds [5], there are some studies on products derived from this exotic species, as reported by [6], who explored using quinoa protein, alone or combined with sodium alginate, to create nanoencapsulations for buriti oil. The developed nanoparticles (Q3 and Q4) showed a marked increase in inhibiting enzymes like α-amylase (inhibition of 88%), α-glucosidase, and amyloglucosidase compared to the unencapsulated oil. In contrast, seeds—the most abundant part of the fruit—are a source of fiber, carotenoids, and phenolic compounds, possessing a high antiradical capacity but low lipid and protein content [7]. MF shows promise in various therapeutic areas, but research is still emerging, especially regarding the shell—a byproduct of fruit pulp extraction. More studies are needed to fully understand the potential of *Mauritia flexuosa* seeds (MFSs) for developing new products for the food and pharmaceutical industries, indicating a need for further exploration and the expansion of its production and processing [4].

Modulating carbohydrate digestion and absorption remains a pivotal therapeutic approach for managing type 2 diabetes mellitus (T2DM) [8]. Central to this process are the enzymes α-amylase and α-glucosidase. α-Amylase, secreted by the salivary glands and pancreas, initiates the hydrolysis of dietary starch into oligosaccharides [9]. Subsequently, α-glucosidase, located in the brush border of the small intestine, breaks down these oligosaccharides into absorbable monosaccharides, primarily glucose. Inhibiting these enzymes delays carbohydrate digestion and glucose absorption, thereby mitigating postprandial hyperglycemia, a critical factor in the management of T2DM. Clinically, α-glucosidase inhibitors (AGIs) such as acarbose, miglitol, and voglibose have been employed to attenuate postprandial glucose spikes [10]. However, their use is often limited by gastrointestinal side effects, including bloating, abdominal discomfort, and diarrhea.

Recent research has focused on identifying natural alternatives with inhibitory activity against these enzymes, aiming for effective glycemic control with fewer adverse effects [11,12]. The therapeutic relevance of targeting these enzymes is further supported by studies indicating that controlling the rate of carbohydrate hydrolysis and glucose absorption can significantly improve glycemic control and insulin sensitivity in patients with type 2 diabetes mellitus (T2DM) [13]. Beyond the specific activities mentioned, Ref. [14] conducted a study to explain the inhibition of α-amylase and α-glucosidase by polyphenols, due to their ability to interact with the enzymes. The search for alternative therapies to manage arterial hypertension, particularly those based on natural products with angiotensin-converting enzyme (ACE) inhibitory activity, remains a pressing research priority. Although current antihypertensive drugs are generally effective, their potential side effects underscore the need for safer, more selective alternatives. Recent advances in computational and biochemical screening have accelerated the discovery of novel α-amylase and α-glucosidase inhibitors from natural sources for diabetes management [15]; similar strategies are proving instrumental in identifying natural ACE inhibitors. Phenolic compounds, for instance, have demonstrated inhibitory activity against carbohydrate-hydrolyzing enzymes, while plant-derived peptides and flavonoids have shown promising ACE inhibitory effects [16]. Crucially, in vitro findings must be substantiated through in vivo studies, such as those employing spontaneously hypertensive rat models, to confirm therapeutic efficacy and physiological relevance [17]. While its pulp is widely used in local food products, the seed or kernel of MF is largely underutilized despite the fact that it is not traditionally consumed and its nutritional value in various forms is unknown. The present study focused on the proximal composition of MFSs and aimed to determine if their ethanolic extract exhibits antidiabetic, anti-inflammatory, and antihypertensive properties. Special emphasis was placed on identifying phytocompounds that indicate therapeutic potential, particularly phenolic compounds, due to their broad spectrum of pharmacological activity. This study utilized a comprehensive bioactivity evaluation approach, incorporating both in vitro and in vivo analyses, to provide a holistic understanding of the pharmacological properties of MFSs.

## 2. Results

The extraction yield was 5% (*w*/*v*). Subsequently, the results described below were obtained.

### 2.1. Proximal and Phytochemical Characterization of MFSs

Table 1 shows the nutritional and chemical composition of MFSs. The percentage of crude protein (9.1%) and fiber (28.4%) was prominent, and only 2.8% of ether extract was detected. Carbohydrates, amino acids, polyphenols, tannins, and flavonoids were detected, while saponins, proteins, terpenes, and alkaloids were undetected under the tested conditions.

Carbohydrates, proteins, polyphenols, flavonoids, and carotenoids were quantified, being the most significant metabolites in the qualitative tests. Polyphenols were the most abundant, with a value of 123.4 µg of gallic acid/100 mg of d.w., followed by flavonoids, carbohydrates, and *β*-carotenoids, which showed values around 0.29 µg/100 mg of dry weight.

### 2.2. Phenolic Composition

Table 2 presents the phenolic profile of MFSs. The evaluated extract was characterized by the presence of various phenols, including p-hydroxybenzoic acid, epigallocatechin gallate (EGCG), quercetin, rosmarinic acid, trans-cinnamic acid, pinocembrin, and rutin, which were commonly found.

In positive-ion-mode LC-MS analysis, compounds such as theobromine, theophylline, caffeic acid, and p-hydroxybenzoic acid were detected with *m*/*z* values of 181.071, 180.066, 179.033, and 137.022, respectively. Isomers, such as theobromine and theophylline, or catechin and epicatechin (both with *m*/*z* = 291.28), were distinguished by their chromatographic retention times. Caffeine and ferulic acid exhibited an *m*/*z* of 195.088, while other acids such as vanillic acid and p-coumaric acid were observed at *m*/*z* = 167.033 and 163.038, respectively, and trans-cinnamic acid at *m*/*z* = 147.043. Flavanols like epigallocatechin (*m*/*z* = 307.079), catechin (*m*/*z* = 291.085), and epicatechin (*m*/*z* = 291.085), along with their gallate derivatives EGCG (*m*/*z* = 459.089) and ECG (*m*/*z* = 443.095), also formed [M+H]^+^ ions. Finally, flavonoids such as quercetin (*m*/*z* = 303.048), luteolin (*m*/*z* = 287.053), naringenin (*m*/*z* = 273.074), apigenin (*m*/*z* = 271.056), and pinocembrin (*m*/*z* = 257.079), as well as ursolic acid (*m*/*z* = 457.366), pelargonidin 3-glucoside (*m*/*z* = 433.111, as its cation), and rutin (*m*/*z* = 611.157), were characterized by their respective protonated molecular ions ([M+H]^+^) derived from their molecular weight plus the addition of a proton.

### 2.3. Antiradical Activity of MFS Ethanolic Extract

Six concentrations of the MFS extract were evaluated against synthetic radicals utilizing DPPH, ABTS, and ORAC assays to determine antioxidant activity. Based on the results obtained, the IC_50_ values are presented in Table 3.

The IC_50_ values obtained indicate that the MFS ethanolic extract exhibits a greater ability to stabilize the ABTS radical, with an IC_50_ value of 171.86 µg/mL. Regarding the stabilization of the DPPH radical, an IC_50_ value of 2989.54 µg/mL was observed. In addition, an IC_50_ value of 363.71 ± 4.2 µg/mL was recorded for the ORAC assay. These results suggest that the metabolites present in the extract exert stabilization through electron donation.

### 2.4. Cell Viability-MTT Assay

The potential cytotoxicity of the MFS ethanolic extract was evaluated at concentrations ranging from 39 to 625 µg/mL. The extract exhibited no signs of cytotoxicity, as cell viability remained above 90% at all concentrations tested, showing no statistically significant difference when compared to the untreated control cells.

### 2.5. Hypoglycemic Activity of MFS Ethanolic Extract

**Figure 1:**
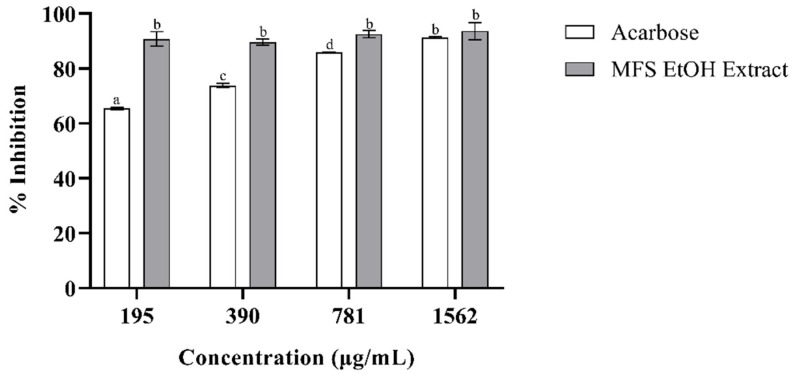
Percentage of inhibition of *α*-amylase enzyme by MFS ethanolic extract. Results presented as mean ± SD (n = 3). The Tukey’s HSD test was used for post hoc multiple comparisons, with statistical significance set at (*p* < 0.05). Treatments that share letters are not significantly different, while treatments with different letters are.

**Figure 2:**
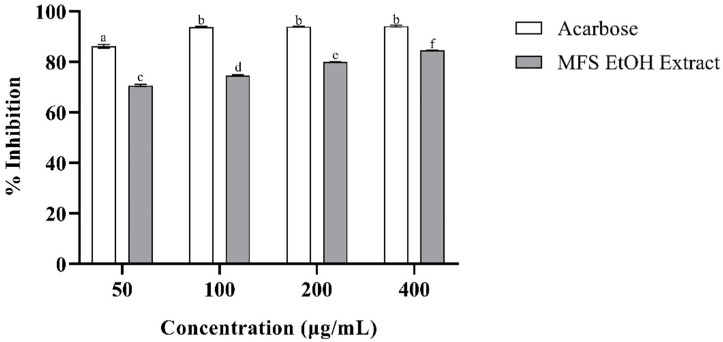
Percentage of inhibition of α-glucosidase enzyme by MFS ethanolic extract. Results presented as the mean ± SD (n = 3). The Tukey’s HSD test was used for post hoc multiple comparisons, with statistical significance set at (*p* < 0.05). Treatments that share letters are not significantly different, while those with different letters are.

In Figure 1 and Figure 2, the results of the inhibition of diabetes-related digestive enzymes are shown.

Figure 1 and Figure 2 present the results concerning the inhibition of the digestive enzymes α-glucosidase and α-amylase, both associated with diabetes, by the MFS ethanolic extract and acarbose, which was used as a positive control. Figure 1 illustrates that acarbose exhibits an inhibition of the α-amylase enzyme directly proportional to its concentration. Conversely, the MFS ethanolic extract demonstrated a high inhibition that was not concentration-dependent and showed no statistically significant differences (*p* > 0.05). However, regarding the inhibition of the α-glucosidase enzyme, differences in the percentage of enzyme inhibition obtained at various concentrations of the MFS ethanolic extract were statistically significant (*p* < 0.05) and concentration-dependent. This contrasts with the behavior of acarbose, where high levels of enzyme inhibition were achieved at concentrations exceeding 100 µg/mL but without a marked dose–concentration effect and no statistically significant differences (*p* > 0.05).

### 2.6. Hypoglycemic Activity by Alloxan Induction

**Figure 3:**
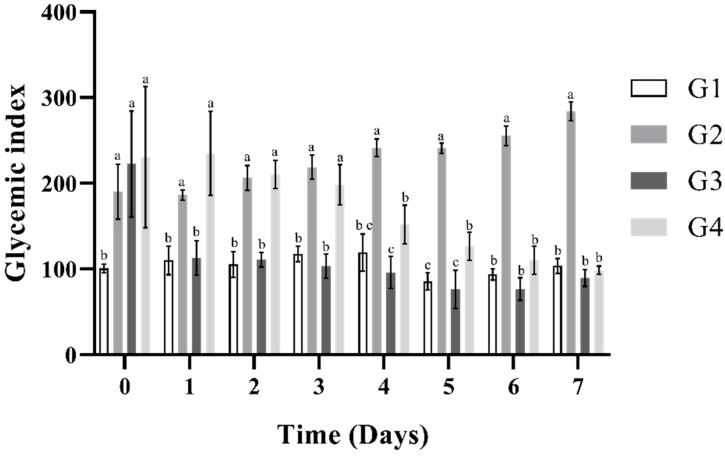
Hypoglycemic activity of MFS ethanolic extract on different experimental groups of Wistar rats. Absolute Control Group G1: Wistar rats without diabetes induction; Negative Control Group G2: Wistar rats with diabetes induction, without treatment; Positive Control Group G3: Wistar rats with induction and glibenclamide (10 mg/kg); G4: Wistar rats with induction and 100 mg/kg of MFS ethanolic extract. The Tukey’s HSD test was used for post hoc multiple comparisons, with statistical significance set at (*p* < 0.05). Treatments that share letters are not significantly different, while those with different letters are.

Figure 3 shows the behavior of blood glucose levels generated by the Absolute Control Group (G1), Negative Control Group (G2), Positive Control Group (G3), and MFS ethanolic extract (G4) treatments. It can be observed that G3, corresponding to the rats that were induced and treated with glibenclamide, showed similar behavior to G1, which consisted of rats without induction or treatment. Regarding the MFS extract (G4), it successfully reduced blood glucose levels, although the values were higher compared to those in G3 (glibenclamide). However, a constant decrease in blood glucose levels was evident throughout the experiment.

### 2.7. Anti-Inflammatory Activity: Auricular Edema in MFS Ethanolic Extract Mice

The inflammatory response varied across the different experimental groups, depending on the treatment administered. In the Absolute Control Group (G1), where no inflammation was induced, no signs of inflammation were observed, serving as the absolute control. In the Negative Control Group 2 (G2), inflammation was induced without subsequent treatment, resulting in comparable levels of inflammation in both ears of each subject, representing the complete inflammatory response.

The Positive Control Group (G3), treated with dexamethasone, a well-established anti-inflammatory agent, exhibited a marked anti-inflammatory effect, with a 94.76% reduction in inflammation compared to the untreated, inflamed group, confirming the efficacy of the drug. Conversely, G4, which received the MFS ethanolic extract after inflammation induction, did not show a statistically significant anti-inflammatory effect. The observed reduction in inflammation was only 7.69%.

### 2.8. Antihypertensive Activity of MFS Ethanolic Extract

#### 2.8.1. Inhibition of Angiotensin-Converting Enzyme (IACE) In Vitro

As shown in Table 4, the MFS ethanolic extract demonstrated concentration-dependent ACE inhibition. At the highest concentration tested (1250 µg/mL), a significant inhibition of 81.90% was achieved. This dose-dependent effect was consistently observed across all evaluated concentrations. The statistical model that best fits the data was $\%IACE=\sqrt{(52,073X}+436.87)$ (*p* < 0.05), which estimates an IC_50_ of 396.19 (218.17–572.32).

#### 2.8.2. In Vivo Antihypertensive Changes in Wistar Rats

**Figure 4:**
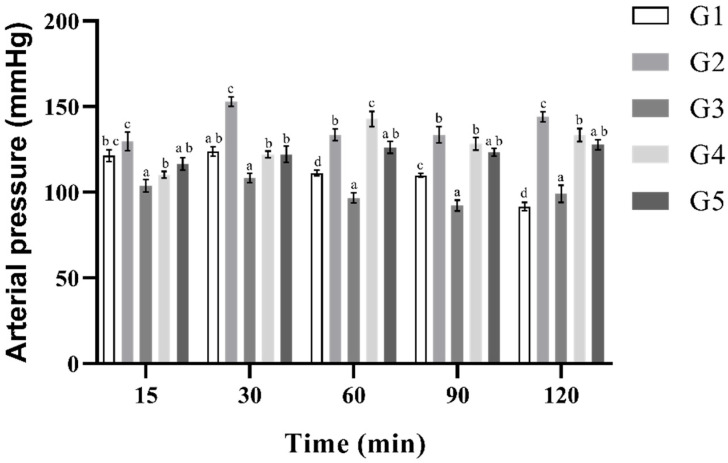
Blood pressure of Wintar rats under different experimental conditions. Absolute Control Group G1: Blank or vehicle (saline solution (i.p.)); Negative Control Group G2: (L-NAME at 10 mg/kg (i.p.)); Positive Control Group G3: (enalapril 10 mg/kg (p.o.) + L-NAME at 10 mg/kg (i.p.)); G4: (MFS EtOH 50 mg/kg (p.o.) + L-NAME at 10 mg/kg (i.p.)); G5: (MFS EtOH 150 mg/kg (p.o.) + L-NAME at 10 mg/kg (i.p.)). The Tukey’s HSD test was used for post hoc multiple comparisons, with statistical significance set at (*p* < 0.05). Treatments that share letters are not significantly different, while those with different letters are.

In Figure 4, the blood pressure changes in rats with L-NAME-induced hypertension over a 120 min period following the administration of various treatments are shown. Group 1 (blank group) exhibited a progressive reduction in blood pressure, from 121.4 ± 1.2 mmHg at 15 min to 91.7 ± 0.8 mmHg at 120 min. In contrast, Group 2 (L-NAME, 10 mg/kg) showed a sustained increase, reaching a peak of 152.9 ± 1.4 mmHg at 30 min while remaining significantly elevated throughout the experiment. Group 3 demonstrated a marked and consistent antihypertensive effect, with blood pressure decreasing from 103.9 ± 1.1 mmHg at 15 min to a minimum of 92.2 ± 1.3 mmHg at 90 min. Group 4 exhibited a dose-dependent response, with an initial rise in blood pressure (up to 142.8 ± 1.6 mmHg at 60 min) followed by a slight decline that did not reach normotensive levels. Meanwhile, Group 5 showed a more stable response, with blood pressure values lower than those of the L-NAME group but still higher than those observed with enalapril. These findings suggest that, while enalapril effectively controls L-NAME-induced hypertension, the MFS extract at 150 mg/mL may exert a modest antihypertensive effect.

## 3. Discussion

This study of MFSs reveals a significant bioactive profile, with notable antioxidant, antidiabetic, and antihypertensive activity. The moderate crude protein and crude fiber contents (9.1% and 28.4%, respectively) are comparable to other members of the Arecaceae family, such as asaí (*Euterpe oleracea*) and buriti (*Mauritia vinifera*), which also exhibit high fiber and bioactive compound contents [18]. However, most studies on Arecaceae species have focused on the fruit pulp, as reported by [19], who confirmed the high fiber content in *Mauritia flexuosa* fruit.

Phytochemical characterization of the MFS ethanolic extract showed the presence of metabolites, with polyphenols being the most predominant, at 123.4 µg of gallic acid per 100 mg of d.w., while the flavonoid concentration was 88.34 ± 1.12. Recent studies have reported higher phenolic contents in MF fruit pulp, as seen in [20], who reported an amount of 378.07 ± 3.12 mg GAE/100 g. Although the difference in total phenolic content is notable, it is essential to highlight that the reported data corresponds to the shell. The results of the chemical analysis support this conclusion. Although various phenolic compounds were detected, the methods used in this study could only specifically identify a few of them, in contrast with the high total phenolic content and antioxidant capacity observed through our assays. Notably, p-hydroxybenzoic acid, one of the identified substances, is a compound known for its antioxidant activity [21] and has also been reported in other species of the Arecaceae family [18].

The antioxidant capacity of the MFS ethanolic extract was notably demonstrated by the stabilization of ABTS, DPPH, and ORAC radicals, exhibiting IC_50_ values of 171.86, 2989.54, and 363.71 µg/mL, respectively. These results indicate a high affinity for electron donation (a characteristic of certain polyphenols present in the extract), thereby confirming the close relationship between phenolic compound concentration and antioxidant activity, as suggested by [22,23]. Moreover, hydroxybenzoic and hydroxycinnamic acids can indirectly enhance cellular antioxidant defenses by activating the Nrf2 signaling pathway, leading to the upregulation of endogenous antioxidant enzymes such as superoxide dismutase (SOD), catalase (CAT), and glutathione peroxidase (GPx), which are crucial for detoxifying reactive oxygen species and mitigating oxidative stress [24]. Furthermore, this same MFS extract exhibited promising antidiabetic properties, which may be attributed to its phenolic content. Nevertheless, the significant concentration of crude fiber and protein identified in the extract opens an interesting avenue of research into the potential of these compounds in future studies involving the whole seed or kernel. Consequently, further investigations are required to validate these observations and elucidate the underlying mechanisms of both activities [25]. The findings of this study demonstrate that the MFS ethanolic extract significantly reduced blood glucose levels in rats with induced diabetes compared to the diabetic control group (*p* < 0.05, unpaired *t*-test). This significant decrease, evidenced by a reduced area under the glucose concentration–time curve (AUC), suggests notable hypoglycemic activity. While less potent than glibenclamide, the observed effect aligns with the existing literature highlighting the beneficial properties of bioactive compounds found in Amazonian fruits. This highlights the potential of the seeds as a valuable natural resource for developing complementary treatments to regulate blood glucose in metabolic conditions such as diabetes.

The potential mechanisms of action observed in our study, such as the reduction in blood glucose levels, may be linked to the inhibition of two key carbohydrate-hydrolyzing enzymes: α-amylase and α-glucosidase. A study by [26] on Arazá extract showed a similar inhibition of the same digestive enzymes and reported the presence of various phenolic compounds such as ellagic, p-coumaric, chlorogenic, and gallic acids [26]; flavonoids such as quercetin, myricetin, and epicatechin; and anthocyanins such as cyanidin-3-*O*-glucoside and malvidin-3-*O*-glucoside [25], some of which were also found in the MFS extract. This suggests that similar phenolic and flavonoid constituents in the MFS extract may contribute to its observed hypoglycemic effects by interfering with carbohydrate digestion and absorption.

Although direct experimental evidence is currently lacking in the literature, the theoretical foundation for investigating this activity is well-established. MF is known to be a rich source of diverse phenolic compounds, including catechin, quercetin, and chlorogenic acid—phytochemicals that have been extensively reported in other plant species to exhibit inhibitory effects against α-amylase and α-glucosidase, which are enzymes that play key roles in glycemic regulation [27,28,29].

In vivo studies have previously shown that the administration of MF extracts or oil improves lipid profiles and enhances antioxidant status [30,31]. These outcomes are consistent with the established biological activities of phenolic compounds [32]. However, it is essential to emphasize that most of these studies have been conducted in non-diabetic models, thereby limiting the interpretation of their direct impact on glucose homeostasis. While the antioxidant properties of phenolic compounds may indirectly contribute to mitigating diabetes-related complications associated with oxidative stress, this mechanism alone does not directly account for reductions in plasma glucose levels.

This context underscores the crucial need for future investigations to assess the α-amylase and α-glucosidase inhibitory potential of *M. flexuosa* extracts, particularly in diabetic in vivo models. Such studies should explore a broader range of concentrations of the ethanolic seed extract, or alternatively, assess the effects of the whole seed. The results would contribute to establishing a more comprehensive and evidence-based understanding of the antidiabetic potential of MF. Concerning glucose regulation, phenolic compounds can influence carbohydrate metabolism through multiple pathways. On one hand, they are capable of modulating glucose absorption by inhibiting key digestive enzymes such as α-amylase and α-glucosidase, which slows the release of monosaccharides and their subsequent entry into the bloodstream. Furthermore, modifying the chemical structure of phenolic compounds—by adding or removing hydroxyl groups (hydroxylation), methyl groups (methylation), or sugar units (glycosylation), or through hydrogenation or galloylation—can either boost or reduce their ability to inhibit α-amylase and α-glucosidase enzymes [33]. Furthermore, some phenolic compounds, such as quercetin and catechin, can directly interact with glucose transporters (GLUTs and SGLTs), altering their expression or activity, which impacts glucose uptake by cells [34].

On the other hand, in terms of cytotoxic activity, the assessment of the ethanolic extract’s effect on leukocytes demonstrated cell viability exceeding 90% across all tested concentrations. This promising safety profile aligns with observations from other palm extracts, such as acaí [35], reinforcing the potential of MFS extracts for various therapeutic and nutritional uses without notable cellular toxicity [36,37,38]. Nevertheless, dedicated in vivo toxicity studies are crucial to thoroughly evaluate the true potential of the seed and its extracts.

Comparatively, the bioactivity observed in MFSs aligns with studies on other closely related species within the Arecaceae family. The reported antioxidant and anti-inflammatory benefits of *Euterpe precatoria* [18] and the hypoglycemic and antioxidant effects of *Acrocomia aculeata* [13], attributed to similar phenolic compounds, further strengthen the rationale for exploring the therapeutic potential of this plant family. The seeds of *Mauritia flexuosa* have been reported to exhibit high antioxidant activity due to the presence of polyphenols, flavonoids, and other phenolic compounds. A study published in 2024 found that seed extracts of *M. flexuosa* demonstrated significant DPPH radical scavenging activity and high total phenolic content [7].

Furthermore, the pulp of *M. flexuosa* is rich in carotenoids, vitamins (A and C), and fatty acids. Research has shown that it is particularly high in vitamin A (beta-carotene) and exhibits strong antioxidant and anti-inflammatory properties [4]. Studies have also indicated that the peel contains a higher polyphenolic content than the pulp, contributing to its potent antioxidant properties. Although less studied, the peel has demonstrated considerable potential for both antioxidant and anti-inflammatory activity [37].

The oil extracted from *M. flexuosa* is rich in oleic and linoleic acids and has shown significant anti-inflammatory activity in in vitro models [38]. In comparison with *M. flexuosa* seed (MFS) extracts and other related species, the pulps of *Euterpe oleracea* (acaí) and *M. flexuosa* are notable for their high antioxidant capacity, largely attributed to anthocyanins in acaí pulp and carotenoids (β-carotene) in *M. flexuosa* pulp. In contrast, *E. oleracea* seeds exhibit lower antioxidant activity [39,40].

With regard to antihyperglycemic and antihypertensive activities, studies on acaí—particularly its pulp and seed extracts—have demonstrated antihyperglycemic effects, including improved insulin signaling and reduced blood glucose levels in animal models, with some evidence from pilot human studies [41,42]. Similarly, the current findings for MFS extract indicate the inhibition of alpha-amylase and alpha-glucosidase enzymes, along with the presence of bioactive compounds such as phenolic acids and flavonoids, which may contribute to metabolic regulation [27,28,29].

Regarding antihypertensive activity, *E. oleracea* seed extract has shown potential to prevent vascular dysfunction and remodeling in hypertensive rats, primarily through antioxidant mechanisms and enhanced nitric oxide production [43,44,45]. These findings align with the antihypertensive effects observed for MFS extract in the present study.

In addition, this study provides compelling preliminary evidence for the antidiabetic potential of the MFS ethanolic extract. The observed hypoglycemic activity, its apparent safety, and supportive findings from related species and their bioactive constituents warrant further comprehensive investigation. Future research should focus on detailed phytochemical characterization, dose–response evaluations, and in vivo studies utilizing the whole seed to fully elucidate its therapeutic efficacy and potential as a complementary strategy for diabetes management [46,47,48].

The observed antihypertensive and antidiabetic potential of the MFS extract appears to be intricately linked to its antioxidant capacity and its influence on key metabolic pathways. In vitro assays consistently demonstrated the extract’s significant ability to neutralize free radicals, exhibiting potent activity in ABTS and DPPH radical scavenging assays. Furthermore, its peroxyl radical scavenging ability, as shown by the ORAC assay, suggests a crucial role in mitigating oxidative stress—a common underlying factor in both diabetes and hypertension pathogenesis. This broad-spectrum radical scavenging capacity likely contributes to the extract’s vascular protective potential by reducing oxidative damage to the endothelium—a vital component for maintaining vascular health and a key factor in the pathophysiology of hypertension.

Specific compounds identified in the MFS extract, such as quercetin and rutin, contribute directly to these observed effects. Quercetin, for instance, significantly influences glucose metabolism by promoting the translocation of GLUT4 to the cell surface in skeletal muscle cells [49]. This process is largely mediated by its ability to stimulate adenosine monophosphate-activated protein kinase (AMPK), a central regulator of cellular energy homeostasis. AMPK activation leads to increased GLUT4 translocation [50], thereby enhancing glucose uptake in an insulin-independent manner. Concurrently, both quercetin and rutin are known to promote the relaxation of vascular smooth muscle, primarily through endothelium-dependent mechanisms, directly contributing to the extract’s antihypertensive properties [51].

The in vitro inhibition of α-amylase and α-glucosidase by the MFS extract, coupled with its observed hypoglycemic effect in alloxan-induced diabetic rats, strongly supports the hypothesis that the extract’s compounds delay carbohydrate digestion and absorption. This, in synergy with enhanced glucose uptake via GLUT4 modulation, underscores a multi-targeted approach to glycemic control. Additionally, preliminary evidence suggesting the extract’s ability to inhibit angiotensin-converting enzyme (ACE) further strengthens its proposed antihypertensive potential by influencing the renin–angiotensin system.

The extract exhibited a high polyphenol content (123.4 µg gallic acid equivalents per 100 mg dry weight), consistent with the findings reported by [52]. In contrast, information regarding the direct analysis of the antioxidant activity of *Mauritia flexuosa* seeds is scarce; however, some studies on fresh seeds have recorded a total phenol content (TPC) of 270.75 mg GAE/100 g after a 96 h extraction period. The highest antioxidant activity (DPPH) was observed after 96 h of dehydration, with results superior to those in this research, indicating the need to standardize drying and extraction procedures to improve the polyphenolic compound content of *M. flexuosa* seeds [53]. These polyphenolic compounds are known to modulate oxidative enzymes, enhance nitric oxide (NO) bioavailability, and promote vascular relaxation [54], which may partially explain the observed biological effects.

In the in vivo model, where hypertension was induced in rats using L-NAME, the administration of the MFS ethanolic extract resulted in a progressive and significant reduction in systolic blood pressure (SBP) compared to the untreated hypertensive group. The antihypertensive effect became evident 60 min post-administration and persisted through the 120 min observation period, suggesting an acute vasodilatory effect. Additionally, the specific phenolic compounds, such as certain plant-derived peptides and flavonoids, can bind to ACE and inhibit its activity, which reduces angiotensin II production and, consequently, promotes vasodilation and a decrease in blood pressure [55].

This behavior may be mediated by the polyphenolic constituents of the extract, which have been widely associated with improved endothelial function and enhanced NO signaling, as reported in other Arecaceae species [56]. Notably, the reduction in SBP observed in animals treated with the MFS extract reached levels comparable to those achieved with the reference drug enalapril, indicating a clear and sustained biological response and highlighting the extract’s potential as a supportive therapeutic agent.

## 4. Materials and Methods

### 4.1. Reagents and Materials

HPLC-grade methanol, water, and formic acid were purchased from Merck. (Darmstadt, Germany) Standard-grade caffeine, theobromine, theophylline, (±)-catechin, (−)-epigallocatechin gallate (EGCG), (−)-epicatechin (EC), (−)-epicatechin gallate (ECG), (−)-epigallocatechin, caffeic acid, p-coumaric acid, rosmarinic acid, quercetin, naringenin, luteolin, kaempferol, pinocembrin, apigenin, cyanidin 3-rutinoside, and pelargonidin 3-glucoside were purchased from Sigma-Aldrich (St. Louis, MO, USA). Antrone, GR for analysis; *β*-carotene synthetic, ≥93% (UV), powder; membrane-permeable yellow dye MTT(3-(4,5-dimethylthiazol-2-yl)-2,5-diphenyltetrazolium bromide; α-glucosidase from *Saccharomyces cerevisiae*; and α-amylase from *Bacillus subtilis* were purchased from Sigma-Aldrich (St. Louis, MO, USA) as well.

### 4.2. Seed Extracts

The seeds of MF ecotype I were obtained from a local cultivator of Florencia-Caqueta, dried in an oven (UN-450, MEMMERT, Schwabach, Germany)at 40 °C for 48 h, and ground into a fine powder. The powdered seeds were then stored for nutritional analysis and extract preparation. The extraction process was performed using ultrasonic-assisted extraction with a cycle of 59 s of sonication followed by 10 s of rest at a power setting of 50 for 15 min. For this procedure, 20 g of dried seeds was cut into small fragments and mixed with a solvent system of ethanol and water (70:30) in a 1:20 (*w*/*v*) ratio. The resulting extracts were concentrated using a rotary evaporator to remove most of the ethanol, and the concentrated extracts were then subjected to lyophilization in a Lyovapor L-200 (BUCHI, Flawil, Switzerland) for subsequent use.

### 4.3. Proximate and Mineral Composition of MFSs

Protein, fat, ash content, and dietary fiber were determined according to the AOAC procedure (2016) [57]. The Macro–Kjeldahl method (N × 6.25) was used to determine the protein content, crude fat was determined using a Soxhlet apparatus, and the sample was extracted with petroleum ether. Ash content was estimated by incineration at 600 ± 15 °C for 5 h, while dietary fiber was analyzed via a gravimetric method. Mineral constituents comprising potassium (K), magnesium (Mg), iron (Fe), calcium, copper (Cu), manganese (Mn), zinc (Zn), and phosphorus (P) were determined by atomic absorption spectrophotometry (SHIMADZU AA-6300, Kyoto, Japan).

### 4.4. Chemical Characterization

The seed extract was characterized using drop-by-drop chemical tests to determine the presence of chemical compounds such as polyphenols (Folin–Ciocalteu test), carbohydrates (Molish and Benedict tests), flavonoids (Shinoda test), terpenes (Lieberman and Salkowski tests), saponins (Foam and Rosenthaler tests), tannins (ferric chloride and gelatin-salt tests), and alkaloids (Tanred, Dragendorff, Valser, and Mayer tests) [22]. The following compound groups were then quantified through spectrophotometric methods using a Thermo Scientific™ Multiskan™ GO Microplate Spectrophotometer (Thermo Fisher Scientific, Waltham, MA, USA) at the wavelengths specified for each method:

Total carbohydrates were quantified using the anthrone method [58]. Total carotenoids were extracted with a hexane-acetone solution, and *β*-carotene was used as a standard to create a calibration curve. Absorbance was measured at 450 nm. Proteins were measured using the Bradford method with a BSA standard, and the measurements were taken at both 590 nm and 450 nm. Total phenols were assessed according to [19], using Folin–Ciocalteu reagent and gallic acid as a standard. Finally, flavonoids were measured at 510 nm with quercetin as a standard, following the test described by [20].

### 4.5. UHPLC-ESI-HRMS Analysis of Phenolic Compounds

The analyzed samples were dissolved in a 1:1 (*v*/*v*) methanol/water mixture with 0.2% (*v*/*v*) formic acid, vortexed for 5 min, and sonicated for 20 min. They were then injected into an Ultra-High-Performance Liquid Chromatograph (UHPLC) Dionex Ultimate 3000 (Thermo Scientific, Sunnyvale, CA, USA) equipped with a binary gradient pump (HP G3400RS, Thermo Scientific, Sunnyvale, CA, USA), an automatic sample injector (WPS 300TRS, Thermo Scientific, Sunnyvale, CA, USA), and a column thermostat unit (TCC 3000, Thermo Scientific, Sunnyvale, CA, USA). The LC-MS interface used electrospray ionization (ESI), and the mass spectrometer was a high-resolution Orbitrap ion current detection system. It was operated in positive mode with a capillary voltage of 3.5 kV. A Hypersil GOLD Aq column (Thermo Scientific, Sunnyvale, CA, USA; 100 mm × 2.1 mm, 1.9 μm particle size) was used. The mobile phase consisted of A-0.1% (*v*/*v*) formic acid and 5 mM ammonium formate in water and B-0.1% (*v*/*v*) formic acid and 5 mM ammonium formate in methanol. The gradient elution started with 100% A, changing linearly to 100% B over 8 min, held for 4 min at 100% B, and returned to the initial conditions in 1 min. The total run time was 13 min, including a 7 min post-run.

Compounds were identified using full scan acquisition mode and the extraction of ion currents (EICs) corresponding to the protonated molecules [M+H]^+^ of the compounds of interest, followed by fragmentation analysis. Quantification of the analytes was based on calibration curves constructed using the certified reference standards listed in the Reagents and Materials section.

### 4.6. In Vitro Bioactivity

#### 4.6.1. Antiradical Activity

ABTS Radical Cation Decolorization Assay

The ABTS assay was based on the method of [59], with slight modifications by [60]. Briefly, a radical solution (3.5 mM ABTS and 1.25 mM potassium persulfate) was prepared in sterile water and left to stand in the dark for 24 h. This solution was then diluted with ethanol at 70% to obtain an absorbance of 0.7 ± 0.02 at 734 nm. The AOX analysis used a 1:49 ratio of extract to radical, with concentrations between 25 and 200 mg/L. The change in optical density was measured using a spectrophotometer (Thermo, Evolution 260, Waltham, MA, USA) at 734 nm after 6 min. The ABTS scavenging capacity of Et-AME was compared with the standard Trolox curve (0.4 to 2.2 µM/L). The equation representing the curve was as follows: (y = −0.2837x + 0.6319R^2^ = 0.9947 (*p* < 0.05)).

AOX was calculated as the percentage of inhibition of absorbance using Equation (1):

(1) $${AOX}_{ABTS}\;=\left(\frac{A_{ABTS}-A_{6min}}{A_{ABTS}}\right)\times 100$$

where *AABTS* is the absorbance of the *ABTS* radical in sterile water and *A*_6*min*_ is the absorbance of the ABTS radical solution mixed with the sample extract/standard. Each sample was measured in triplicate. The mean and standard deviation were calculated.

DPPH Radical Cation Decolorization Assay

The methodology proposed by [61] was followed with some modifications. A 0.5 mL aliquot of a 0.02% DPPH solution, in ethanol (99%), was added to 0.5 mL of each Et-AME. The mixture was stored in the dark (30 min), and the absorbance at 517 nm against a blank (solvent and DPPH) was measured. The standard curve was prepared with Trolox (0.1 to 2.0 μM/L). The equation representing the curve was as follows: (y = −0.0947x + 0.2229R^2^ = 0.9906 (*p* < 0.05)). The percentage of inhibition of *DPPH* of the test sample and known solutions of Trolox were calculated using Equation (2):

(2) $${AOX}_{DPPH}\;=\left(\frac{A_{0}-A}{A_{0}}\right)\times 100$$

where *A*_0_ is the absorbance of the *DPPH* radical in ethanol (blank) and *A* is the absorbance of the *DPPH* radical solution mixed with the sample extract/standard. Each sample was measured in triplicate. The mean and standard deviation were calculated.

ORAC Assay

The determination of total antioxidant capacity by oxygen radical absorption (ORAC) was carried out according to [62]. The standard reference curve was constructed using different concentrations of Trolox (1 to 100 µM/L). The equation representing the curve was as follows: (y = 183,242x + 5 × 10^6^R^2^ = 0.9951 (*p* < 0.05)).

Cell Viability Using MTT Assay

The blood used for the test was obtained from a healthy 26-year-old male volunteer, with prior signing of informed consent, in compliance with the ethical principles for studies with human samples. For this, leukocytes were separated following the protocol of [63], while concentrations of MFS ethanolic extract between 39 and 625 μg/mL were used. For the assay, microplates were used: 25 μL of leukocytes, 25 μL of extract, and 50 μL of MTT were mixed and incubated at 37 °C for 1 h. After this, DMSO (0.5% *w*/*v*) [64] was added and stirred for 10 min to dissolve the formazan crystals. Finally, the microplates were read at 570 nm. This assay was performed to assess the viability of the cells [65]. The negative control (untreated cells) consisted of leukocytes incubated with MTT in the absence of the extract; this group represented 100% cell viability. A baseline control of cells incubated without MTT was also included to assess initial cell health. Each sample was measured in triplicate.

#### 4.6.2. Hypoglycemic Activity

Inhibitory Activity of α-Glucosidase Enzyme

The α-glucosidase enzyme (0.075 units) was mixed with the extract at different concentrations (50–200 μg/mL). Subsequently, 3 mM of p-nitrophenyl glucopyranoside (pNPG) was added as a substrate, and the mixture was homogenized to initiate the reaction [66]. The mixture was then incubated at 37 °C for 30 min, and the reaction was stopped by adding 2 mL of Na_2_CO_3_. α-glucosidase activity was determined by measuring the release of p-nitrophenol from pNPG at 400 nm [67]. The inhibitory activity of α-glucosidase was calculated using Equation (3):

(3) $$\%\;Inhibition=\left(\frac{Abs\;Control-Abs\;Samples}{Abs\;Control}\right)*100$$

Inhibitory Activity of α-Amylase Enzyme

The α-amylase enzyme was mixed with different extract concentrations (50–200 μg/mL). A 0.5% starch solution was added as a substrate to initiate the reaction. The reaction mixture was incubated at 37 °C for 5 min and then terminated by adding 2 mL of DNS reagent (3,5-dinitrosalicylic acid). The reaction mixture was then heated for 15 min at 100 °C and then diluted with 10 mL of distilled water in an ice bath [66]. Finally, α-amylase activity was determined by measuring the absorbance at 540 nm [67]. The inhibitory activity of α-amylase was calculated using Equation (4):

(4) $$\%\;Inhibition=\left(\frac{Abs\;Control-Abs\;Samples}{Abs\;Control}\right)*100$$

Hypoglycemic Activity by Alloxan Induction in Wistar Rats

Experiments to determine in vivo antidiabetic activity were conducted in an appropriate animal experimentation laboratory at the University of Tolima, following the protocol of [68].

All in vivo experiments in this research adhered to the ethical guidelines established in Resolution No. 008430 of 1993 from the Ministry of Health and Law 84 of 1989 of the Republic of Colombia. Similarly, the research was submitted to the Ethics Committee of the Faculty of Sciences at the University of Tolima and was approved for execution under Act 02 of October 2019.

Wistar male rats, normoglycemic, with an approximate weight of 220 g, were used. All animals were housed in the laboratory with access to water and food ad libitum. When they reached an average weight, diabetes was induced via intraperitoneal injection of hydrated alloxan (100 mg/kg b.w.). Blood glucose levels of the experimental animals were checked two days after the initial measurement. A commercial glucometer, GlucoQuick G30a (Acon Laboratories, Inc., San Diego, CA, USA), was used to determine glucose levels in the rats. Blood samples were collected by puncturing the tip of the tail, discarding the first drop, and collecting the subsequent drop on a reactive strip. After confirming the successful induction of diabetes, treatments were administered. The experimental groups are listed in Table 5.

#### 4.6.3. Antihypertensive Activity

Inhibition of ACE

For the analysis, 40 μL of each sample was added to 100 mL of HHL substrate (prepared in 0.1 M sodium borate buffer, with 0.3 M NaCl at pH 8.3). Subsequently, 2 mU of the ACE (EC 3.4.15.1) with a specific activity of 5.1 U/mg, dissolved in 50% glycerol, was added. The reaction was carried out at 37 °C for 30 min. The enzyme was deactivated by lowering the pH, via the addition of 150 mL of 1 N HCl. The formed hippuric acid was extracted with 1000 μL of ethyl acetate. After agitation and subsequent centrifugation at 4000× *g* for 10 min at room temperature, 750 μL of the organic phase was taken. This volume was evaporated by heating at 95 °C for 15 min. The residue of hippuric acid was dissolved in 800 μL of distilled water, and after shaking, it was measured at 228 nm. The ACE activity was calculated as the amount of soluble protein needed to inhibit 50% of the enzyme. The activity of each sample was determined in triplicate.

% IECA = ((Abs control − Abs samples)/(Abs control − Abs blank)) × 100

where Abs control denotes the absorbance of hippuric acid formed after the action of ACE without an inhibitor, Abs blank is the absorbance of HHL that did not react and was extracted with ethyl acetate, and Abs samples represents the absorbance of hippuric acid formed after the action of ACE in the presence of inhibitory substances. This spectrophotometric assay utilized the properties of angiotensin-converting enzyme derived from rabbit lung [69].

Measurement of Effects on Systolic Blood Pressure in Wistar Rats

Experimental animals

Male Wistar rats, aged 9 to 11 weeks and weighing between 180 and 220 g, were used in this study. The animals were supplied by the Animal Facility of the Department of Pharmacy at the National University of Colombia. The rats were housed under standardized conditions of controlled temperature and 12 h light/dark cycles. They had free access to water and food, except on the day of the experimental intervention, when they underwent a controlled 12 h fasting period.

Systolic blood pressure was measured indirectly in the tail of rats, following the protocol described in [64,70], with specific adaptations for this study. The conditioning protocol began two weeks prior to the experimental induction of hypertension, during which daily blood pressure measurements were performed using a non-invasive ultrasound transducer (PANLAB LE 5002, Panlab, S.L., Barcelona, Spain). This device enables the precise measurement of blood pressure in rats using the tail-cuff method.

After the conditioning period, a significant reduction in the animals’ stress levels was observed, facilitating easier handling during the measurements and improving the accuracy of the data obtained. Subsequently, the rats were randomly assigned to experimental treatment groups (n = 6) according to the experimental design shown in Table 6.

After a 12 h fasting period, the animals underwent intraperitoneal induction with L-NAME. Subsequently, the controls (both positive and negative) and the treatments (extracts and protein) were administered. A follow-up study was conducted to measure the blood pressure of each animal over a two-hour period and assess the efficacy of the treatments in mitigating the increase in blood pressure induced by L-NAME.

Systolic blood pressure was determined using the tail-cuff method, which functions by occluding blood flow with a cuff and utilizing a transducer to monitor pulse variations throughout the measurement. The measurement was performed by placing the animal in a rat restrainer (after a one-week acclimation period in the restrainer), maintaining a constant temperature of 29–32 °C to induce tail dilation. A cuff was then positioned at the base of the tail, and a non-invasive ultrasonic transducer (PANLAB-LE 5002, a non-invasive blood pressure monitor, Panlab, S.L., Barcelona, Spain) was used to capture the pulse signal and blood pressure [64,70].

#### 4.6.4. Anti-Inflammatory Activity: Auricular Edema in Mice

Auricular edema was induced by administering 13-acetate-12-O-tetradecanoylphorbol (TPA) according to the methodology described by [71] and with modifications proposed by [72]. The experimental groups are listed in Table 7.

The treatments and the positive control were administered to each corresponding experimental group at a dose of 1 mg per ear. After the treatment was applied to all individuals within a group, a TPA solution (2.5 μg) dissolved in acetone (20 μL) was topically applied to both the internal and external surfaces of each mouse’s right ear at a rate of 10 μL per side to induce inflammation.

Four hours after the application of TPA, the mice were sacrificed by cervical dislocation, and discs (7 mm in diameter) were obtained from the right (treated) and left (untreated) ears using a punch. The discs were weighed to determine the edema generated via calculating the weight variation (delta) (Pt-Pnt).

### 4.7. Statistical Analysis

Data were analyzed by a one-way analysis of variance (ANOVA) to compare mean values among concentrations. Tukey’s HSD test was used for post hoc multiple comparisons, with statistical significance set at *p* < 0.05. Additionally, a probabilistic model was fitted to determine the half-maximal inhibitory concentration (IC50) using STATGRAPHICS Centurion XV (v. 15.2.06). All in vitro experiments were performed in triplicate. For the in vivo experiments, each animal corresponded to a specific treatment, following the methodology previously described. The statistical analysis applied to these in vivo results also followed the same methods as previously described. Figures were generated with OriginPro 2023 (v. 10.0.0.244).

## 5. Conclusions

This study demonstrates that MFSs, derived from ecotype I fruits and constituting the most significant proportion of the fruit’s biomass, are a promising source of various chemical compound groups despite their general underutilization. Notably, they exhibit a considerable amount of crude fiber and phenolic compounds and an average protein content. Furthermore, the ethanolic extract of MFSs yielded compounds with promising hypoglycemic potential in vitro against digestive enzymes such as alpha-amylase and alpha-glucosidase—key targets in diabetes management. This potential was verified in vivo in Wistar rats with induced diabetes subsequently treated with 100 mg/kg of MFS extract, evidenced by a significant reduction in blood glucose levels. Although this hypoglycemic effect was less potent than that of glibenclamide, it is likely mediated by the inhibition of these digestive enzymes and is related to the content of phenolic compounds, including polyphenols and flavonoids. Additionally, the ethanolic extract of MFSs showed significant ACE inhibition at moderate concentrations and a moderate antihypertensive effect at a concentration of 150 mg/kg. Taken together, these findings support the potential of the ethanolic extract of *Mauritia flexuosa* seeds as a promising natural source of antioxidant and antihypertensive agents. Its effects may contribute to the development of complementary strategies for managing arterial hypertension. However, further studies are necessary to isolate and identify the active compounds, elucidate their specific mechanisms of action, and assess long-term effects through subchronic and chronic toxicity studies. These findings represent the first specific report in the literature regarding these effects, which aligns with the known benefits of bioactive compounds in Amazonian fruits and the Arecaceae family. While suggesting MFSs as a potential natural adjunct for diabetes management, further research is essential to identify specific active compounds, establish dose–response relationships, and explore the benefits of the whole seed through in vivo assays. Therefore, complementary studies should be developed in the future to fully demonstrate the potential of this part of the MF fruit as a complementary therapeutic strategy for diabetes and hypertension management.

## Figures and Tables

**Table 1:** Bromatological and phytochemical characterization of MFSs and ethanolic extract of MFSs. + = low; − = absence. Carbohydrates, polyphenols, flavonoids and *β*-carotenoids. Results are expressed as µg equivalents of glucose, gallic acid, quercetin, and beta-carotene, respectively, per 100 mg dry weight (d.w.).

Parameters	Results	Metabolite	Test	Results
% Ash	2.6	Carbohydrates	Antrona	6.15 ± 0.5
% Crude protein	9.1		Molish	+
Ethereal extract	2.8		Benedict	−
% Crude fiber	28.4	Saponins	Foam	−
% Nitrogen			Rosenthaler	−
% Ca	0.78	Amino acids	Ninhidrin	+
% Mg	0.12	Protein and peptides	Biuret	−
Na (mg/kg)	394.4	Polyphenols	Folin-Ciocalteu	123.4 ± 7.21
% K	0.45	Tannins	Ferric chloride	−
Fe (mg/kg)	13.9		Gelatin-salt	−
Cu (mg/kg)	1.8	Flavonoids	Shinoda	88.34 ± 1.12
Mn (mg/kg)	11.0		Zn/HCl	+
Zn (mg/kg)	2.3	Terpenes	Lieberman	
B (mg/kg)	84.0		Salkowski	
% P	0.72	Alkaloids	Dragendorff	−
% S	11.21		Tanred	−
			Valser	−
			Mayer	−
		*β*-Carotenoids	spectrophotometric	0.29 ± 0.9

**Table 2:** UHPLC-ESI-HRMS identification and quantification of phenolic compounds in MFS.

Compound	RT (min)	LOQ mg/kg	[M+H]^+^ (*m*/*z*)	Concentration (mg/kg)
Theobromine	3.1	0.1	181.071	<0.1
Theophylline	3.7	0.1	180.066	<0.1
Epigallocatechin (EGC)	3.7	0.1	307.079	<0.1
Catechin	3.7	0.1	291.085	<0.1
Epicatechin (EC)	4.2	0.1	291.085	<0.1
p-Hydroxybenzoic Acid	3.8	0.4	137.022	2.2
Caffeine	4.1	0.1	195.088	<0.1
Caffeic Acid	4.2	0.1	179.033	<0.1
Vanillic Acid	4.6	0.1	167.033	<0.1
Epigallocatechin Gallate (EGCG)	4.1	0.2	459.089	<0.2
p-Coumaric Acid	4.7	0.1	163.038	<0.1
Epicatechin Gallate (ECG)	4.6	0.1	443.095	<0.1
Ferulic Acid	5.2	0.1	195.086	<0.1
Quercetin	5.8	0.1	303.048	0.2
Rosmarinic Acid	5.1	2.5	359.074	<2.5
Luteolin	6.0	0.1	287.053	<0.1
Trans-Cinnamic Acid	5.8	0.4	147.043	<0.4
Naringenin	5.8	0.1	273.074	<0.1 *
Apigenin	6.3	0.1	271.058	<0.1 *
Pinocembrin	6.6	0.1	257.079	0.3
Ursolic Acid	9.0	0.1	457.366	<0.1
Pelargonidin 3-glucoside	4.3	0.1	433.111	<0.1
Rutin	5.2	0.1	611.157	0.5

LOQ: Limit of quantification. * Detected below the minimum quantification level.

**Table 3:** IC_50_ of MFS ethanol extracts to inhibit DPPH, ABTS, and ORAC radicals.

Assay	IC_50_ (µg/mL)
DPPH	2989.54 ± 3.5 a
ABTS	171.86 ± 3.1 b
ORAC	363.71 ± 4.2 c

Results presented as mean ± SD (n = 3). Treatments that share letters are not significantly different, while treatments with different letters are.

**Table 4:** Percentage of inhibition of ACE at different concentrations of MFS ethanolic extract.

MFS Et-OH (µg/mL)	% IACE
1250	81.90 ± 0.19 a
625	65.65 ± 0.10 b
312.5	40.71 ± 0.19 c
156.25	38.84 ± 0.04 d
78.12	24.42 ± 0.14 e

Results presented as mean ± SD (n = 3). Treatments that share letters are not significantly different, while treatments with different letters are.

**Table 5:** Experimental groups of hypoglycemic activity by alloxan induction in Wistar rats.

Group	Induction (Intraperitoneal Administration)	Experimental Treatments (Oral Administration)	n
G1 (Vehicle)	Normoglycemic rats	1 mL of physiological saline	6
G2 (Negative control)	Rats with alloxan-induced diabetes	1 mL of physiological saline	6
G3 (Positive control)	Rats with alloxan-induced diabetes	1 mL of glibenclamide 10 mg/kg	6
G4 (Treatment)	Rats with alloxan-induced diabetes	100 mg/kg of MFS ethanolic extract	6

**Table 6:** Experimental groups in the antihypertensive evaluation of Wistar rats.

Group	Treatments	n
Group 1 (Vehicle)	Solución Salina Fisiológica (i.p.)	6
Group 2 (Negative control)	L-NAME a 10 mg/kg (i.p.)	6
Group 3 (Positive control)	Enalapril 10 mg/kg (p.o.) + L-NAME a 10 mg/kg (i.p.)	6
Group 4 (Treatment)	MFS ethanolic extract 50 mg/kg (p.o.) + L-NAME a 10 mg/kg (i.p.)	6
Group 5 (Treatment)	MFS ethanolic extract 150 mg/kg (p.o.) + L-NAME a 10 mg/kg (i.p.)	6

(Oral administration: p.o.; Intraperitoneal administration: i.p.).

**Table 7:** Treatments to determine percentage of anti-inflammatory activity.

Group	Treatments (Topical Administration)	Induction (Topical Administration)	n
G1(Vehicle)	10 μL of physiological saline	Mice without TPA induction	6
G2 (Negative control)	10 μL of physiological saline	Mice with TPA induction	6
G3 (Positive control)	10 μL of dexamethasone (1 mg/ear)	Mice with TPA induction	6
G4 (Treatment)	10 μL MFS ethanolic extract (1 mg/ear)	Mice with TPA induction	6

## Data Availability

Data are contained within the article.

## Abbreviations

The following abbreviations are used in this manuscript:

MF	*Mauritia flexuosa*
MFS	*Mauritia flexuosa* seeds
EGCG	Epigallocatechin Gallate
EC	Enzyme Commission number
DPPH	2,2-diphenyl-1-picrylhydrazyl
ABTS	2,2′-azino-bis(3-ethylbenzothiazoline-6-sulfonic acid)
BSA	Bovine serum albumin
DMSO	Dimethyl sulfoxide
